# Supplementary material for: CYK4 relaxes the bias in the off-axis motion by MKLP1 kinesin-6
Source: Commun Biol. 2021 Feb 10;4:180. doi: 10.1038/s42003-021-01704-2 (PMC7876049; doi:10.1038/s42003-021-01704-2)
Supplement: Supplementary file 1 — Supplementary Information [file 42003_2021_1704_MOESM1_ESM.pdf]

Supplementary information for

CYK4 relaxes the bias in the off-axis motion by MKLP1 kinesin-6

Yohei Maruyama[1], Mitsuhiro Sugawa[1,2], Shin Yamaguchi[1], Tim Davies[3,6], Toshihisa Osaki[4], Takuya Kobayashi[1], Masahiko Yamagishi[1,2], Shoji Takeuchi[4,5], Masanori Mishima[3*], Junichiro Yajima[1,2,5*]

[1] *Department of Life Sciences, Graduate School of Arts and Sciences, The University of Tokyo, Meguro-ku, Tokyo 153-8902, Japan*

[2] *Komaba institute for Science, The University of Tokyo, Meguro-ku, Tokyo 153-8902, Japan*

[3] *Centre for Mechanochemical Cell Biology and Division of Biomedical Sciences, Warwick Medical School, University of Warwick, Coventry CV4 7AL, UK*

[4] *Institute of Industrial Science, The University of Tokyo, Meguro-ku, Tokyo 153-8505, Japan*

[5] *Research Center for complex Systems Biology, The University of Tokyo, Meguro-ku, Tokyo 153-8902, Japan*

[6]*Present address: Department of Biosciences, Durham University, Durham DH1 3LE, UK*

[*]Correspondence should be addressed to J.Y. (yajima@bio.c.u-tokyo.ac.jp) or M.M. (M.Mishima@warwick.ac.uk).

Supplementary Figures

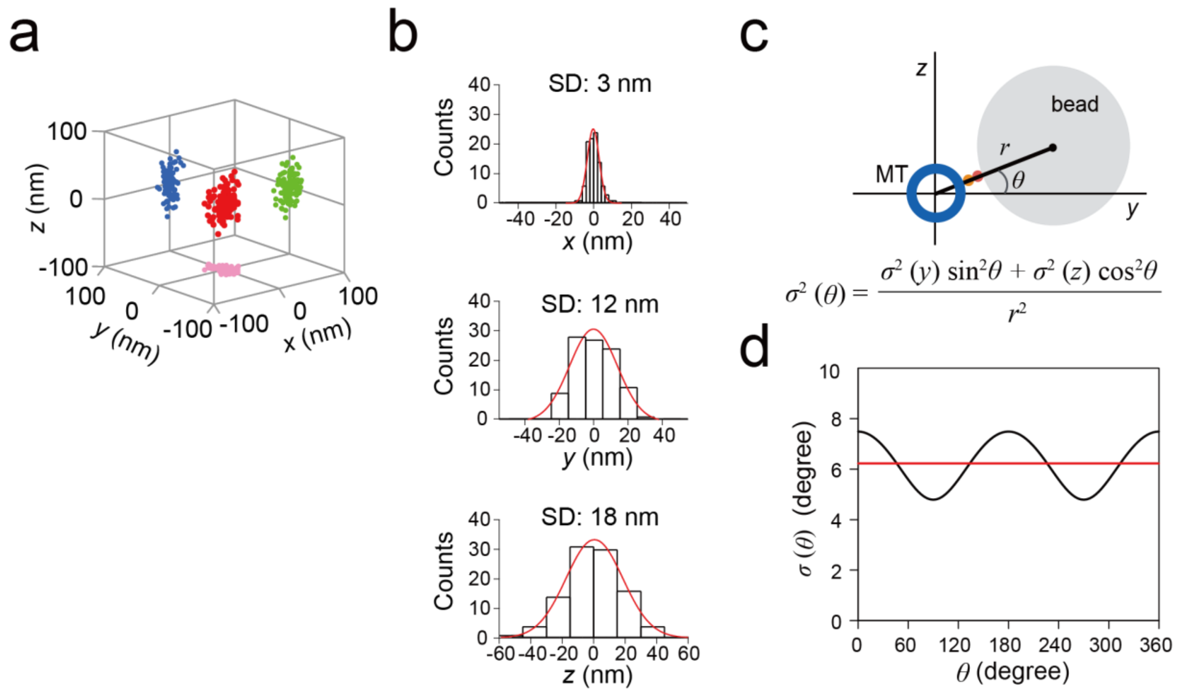

Supplementary Fig. 1: The distributions of the kinesin-coated bead positions on the suspended microtubules.

a The 3D plot shows the positions of a 0.22 μm diameter M_2-coated bead fixed on a suspended microtubule in the presence of AMPPNP during a 10 second recording at 10 frames per second. X-y-z (red), x-y (pink), x-z (blue), and y-z (green) plots are shown. **b** Histograms of the x, y and z positions of the 0.22 μm bead. The standard deviations were derived from a Gaussian fitting. **c, d** Estimation of the precision in angle determination. **c** Schematic of the geometry of the suspended microtubule and the bead. Note that this is not to scale and that only a single motor interacting with the microtubule is illustrated for simplicity. According to the general law of error propagation, when there is no correlation between the errors in the Cartesian coordinates y and z, the standard deviation of the angle θ, $\sigma(\theta)$, can be related to r, θ, and the standard deviations of y and z, $\sigma(y)$ and $\sigma(z)$, respectively, via the formula shown in this panel. Considering the geometry of the system, we can assume that the distance between the center of the bead and the microtubule axis, r, is largely constant. Based on the radii of the microtubule (blue, ~12.5 nm) and the bead (gray, ~110 nm), and the sizes of the motor construct (orange, ~10 nm) and the antibody (red, ~5 nm), we estimate the mean r to be ~ 140 nm. **d** The standard deviation of θ calculated with r = 140 nm is plotted against θ (black curve) with an average value of 6.3 degrees (red line). The range of the precision of the angle (5 ~ 7.5 degree) is much smaller than the angle between two protofilaments (360 degrees / 13 = 28 degrees or 360 degrees / 14 = 26 degrees).

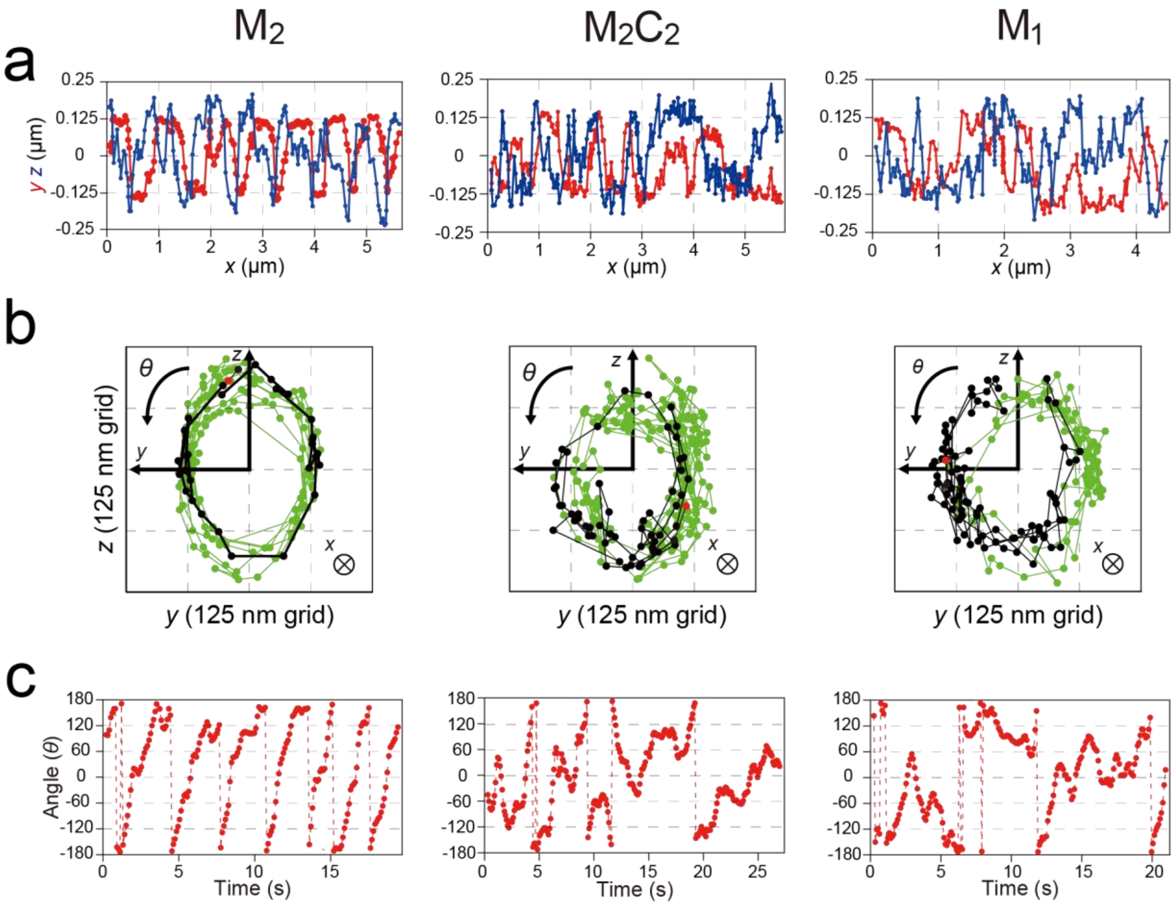

Supplementary Fig. 2: The 3D trajectories of M_2-, M_2C_2-, and M_1-coated beads moving along suspended microtubules.

a The x-y (red) and x-z (blue) trajectories of M_2- (left), M_2C_2- (middle), and M_1- (right) coated bead movement along the suspended microtubule. Images were recorded at 0.1 s intervals. **b** The y-z trajectory of the bead movement shown in (**a**). The trajectory of the first revolution is shown by the black line and begins at the red dot. The trace shows left-handed helical movement of the M_2- (left), M_2C_2- (middle), and M_1- (right) coated bead along the microtubule. **c** The rotation angle on the y-z plane was plotted against time. The y-z trajectory of the bead movement shown in (**b**) was smoothed using a rolling 3-frame average filter and then the rotation angle was plotted. The periodical pattern of the angle (θ) indicates that the left-handed helical motion was smooth for M_2-coated bead (left). For M_2C_2- (middle) and M_1- (right) coated bead, the angles (θ) were not periodically increased per complete rotation, indicating that the left-handed helical motion was less smooth and more fluctuating than that driven by M_2.

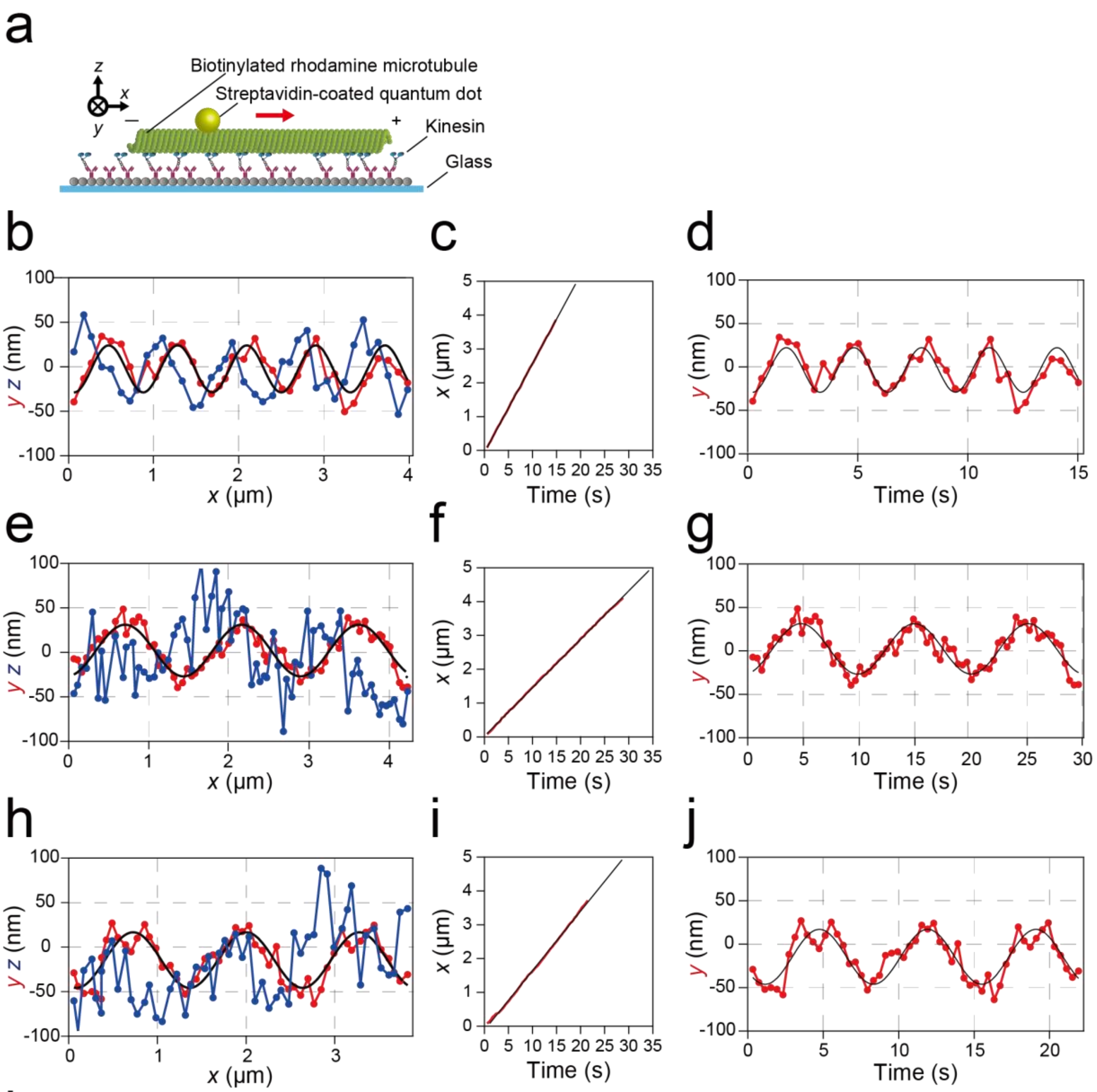

k

	Pitch (μm)	Longitudinal velocity (μm s^{-1})	Rotational velocity (rev s^{-1})
M_2	0.8 ± 0.1 (*N* = 15)	0.27 ± 0.01 (*N* = 15)	0.33 ± 0.03 (*N* = 15)
M_2C_2	1.6 ± 0.4 (*N* = 17)	0.14 ± 0.01 (*N* = 17)	0.10 ± 0.02 (*N* = 17)
M_1	1.4 ± 0.7 (*N* = 15)	0.19 ± 0.01 (*N* = 15)	0.18 ± 0.12 (*N* = 15)

Supplementary Fig. 3: The 3D trajectories of the corkscrew motion of gliding microtubule driven by MKLP1 constructs.

a A schematic illustration of the microtubule corkscrewing assay. The sparsely biotinylated, rhodamine-labeled microtubule with a QD (λ = 525 nm) attached is sliding and rotating driven by the motors anchored to the Protein G (gray)-coated glass surface via anti-His-tag antibody (purple). Images were recorded at 0.4 s intervals. **b** The *x*-*y* (red) and *x*-*z* (blue) trajectories of corkscrewing microtubules driven by M_2. The rotational pitch of corkscrewing microtubules driven by M_2 was determined by fitting the *x*-*y* position of the QD with a sine function (black line), yielding a value of

0.8 µm. **c** Time course of *x*-displacement of the microtubule motion shown in (**b**). The longitudinal velocity was determined by fitting the *x*-t position of the QD with a linear function (black line), yielding a value of 0.26 µm s^{-1}. **d** Time course of *y*-displacement of the microtubule motion shown in (**b**). The rotational velocity was calculated by taking the reciprocal of rotation time period, which was determined by fitting the *y*-t position of the QD with a sine function (black line), yielding a value of 0.33 rev s^{-1}. **e** The *x*-*y* (red) and *x*-*z* (blue) trajectories of corkscrewing microtubules driven by M_2C_2. The value of rotational pitch was 1.5 µm. **f** Time course of *x*-displacement of the microtubule motion shown in (**e**). The value of longitudinal velocity was 0.14 µm s^{-1}. **g** Time course of *y*-displacement of the microtubule motion shown in (**e**). The rate of rotation was 0.10 rev s^{-1}. **h** The *x*-*y* (red) and *x*-*z* (blue) trajectories of corkscrewing microtubules driven by M_1. The value of rotational pitch was 1.3 µm. **i** Time course of *x*-displacement of the microtubule motion shown in (**h**). The value of longitudinal velocity was 0.18 µm s^{-1}. **j** Time course of *y*-displacement of the microtubule motion shown in (**h**). The rate of rotation was 0.14 rev s^{-1}. **k** A summary of the rotational pitch, longitudinal velocities, and rotational velocities of the microtubule corkscrewing motion driven by the MKLP1 constructs. Errors are standard deviation (SD). *N* means the number of microtubules.

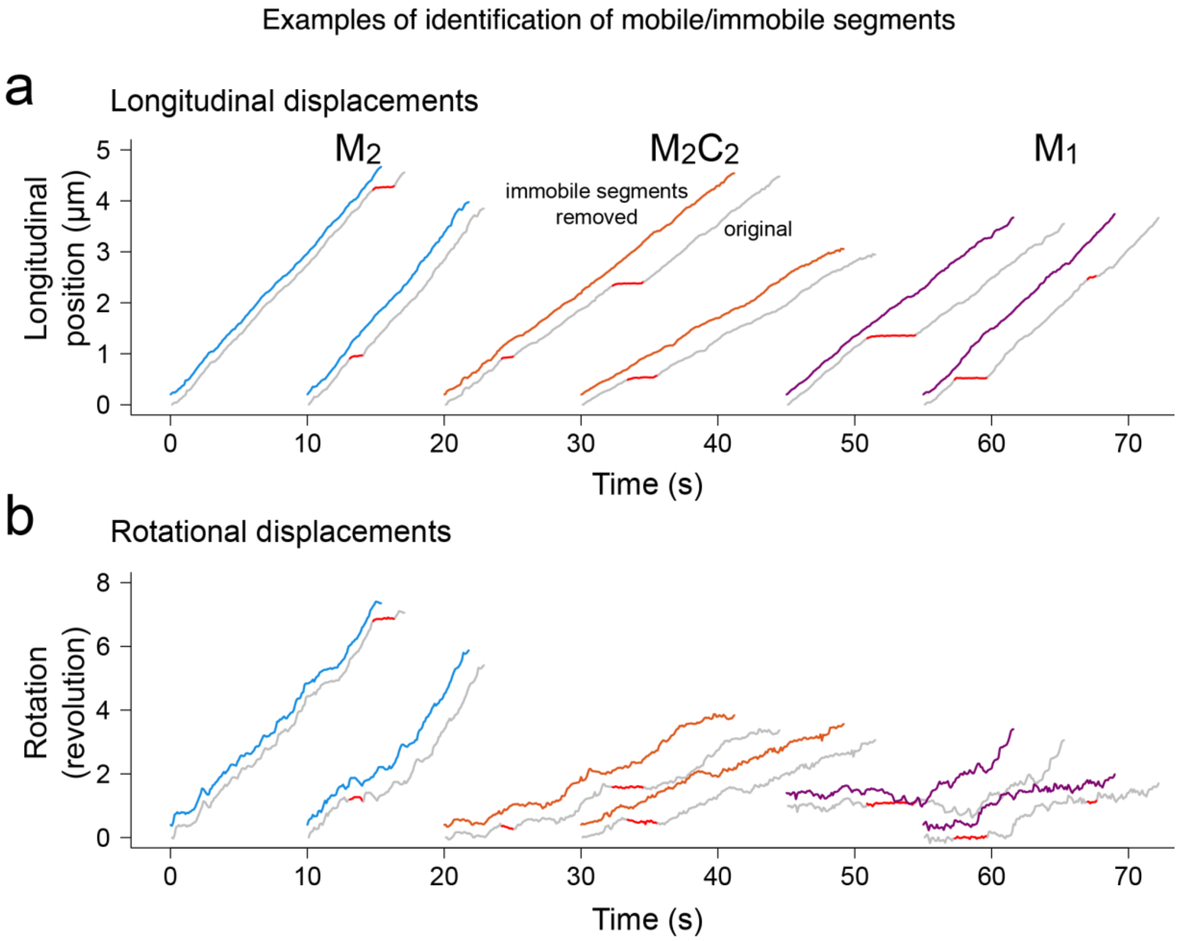

Supplementary Fig. 4: Examples of identification of mobile/immobile segments.

a Longitudinal displacements as already shown in Fig. 4g. **b** Rotational displacements. Typical examples of the trajectories that were inferred to have an immobile segment (red). The segments inferred to be in the mobile mode (gray) were reassembled to generate a virtual trajectory consisted of the displacements uniformly in the mobile mode (blue: M_2, orange: M_2C_2, purple: M_1).

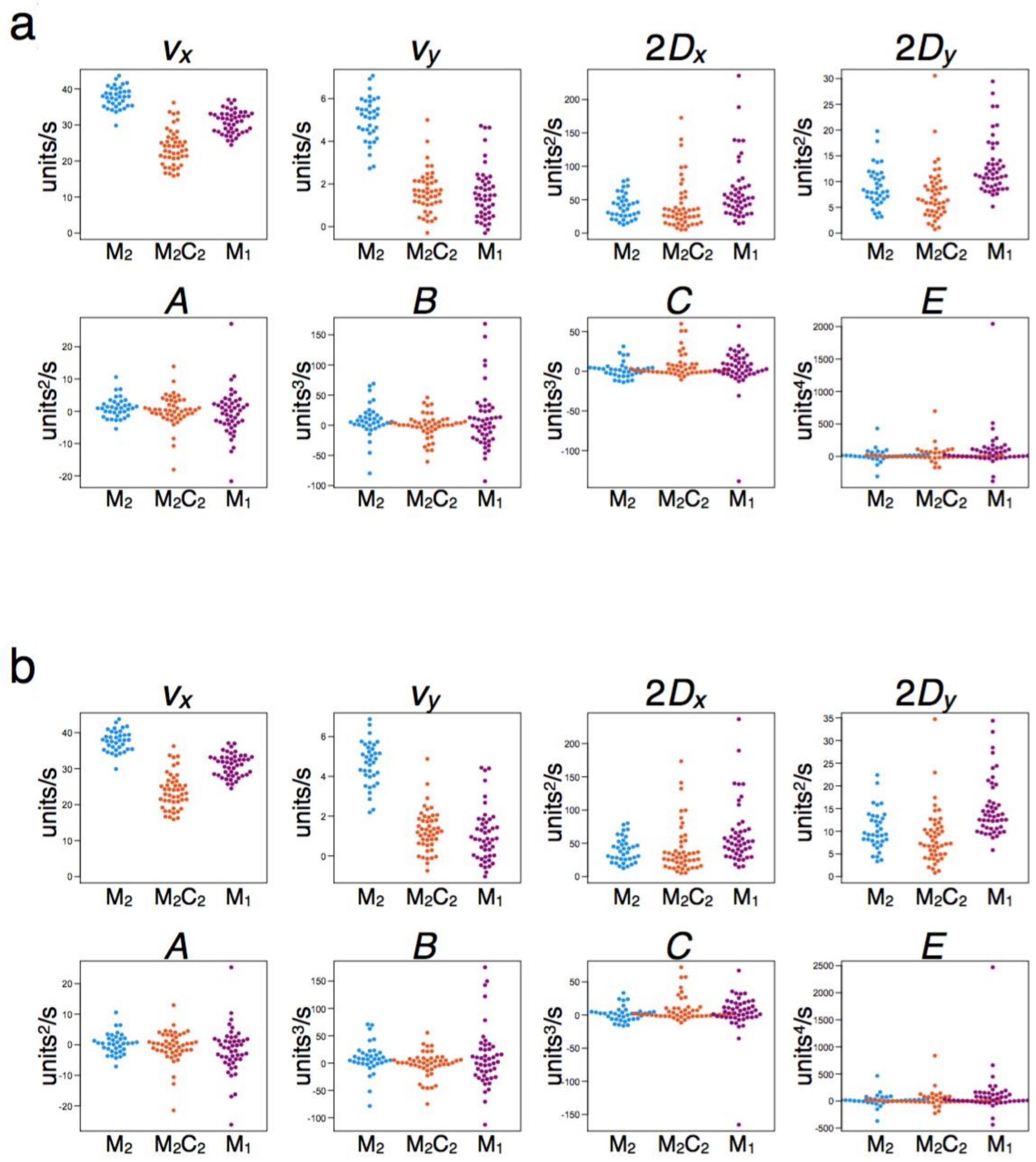

Supplementary Fig. 5: Macroscopic drift and diffusion parameters estimated for individual trajectories.

Displacements on the 2D microtubule lattice surface were calculated by mapping the trajectory data onto the 13 (**a**)- or 14 (**b**)-protofilament microtubule model. The parameters, v_x, v_y, $2D_x$, $2D_y$, A, B, C, and E, were calculated based on the CVE-based approach ("Inference of the hopping rates and preference" section in Materials and Methods).

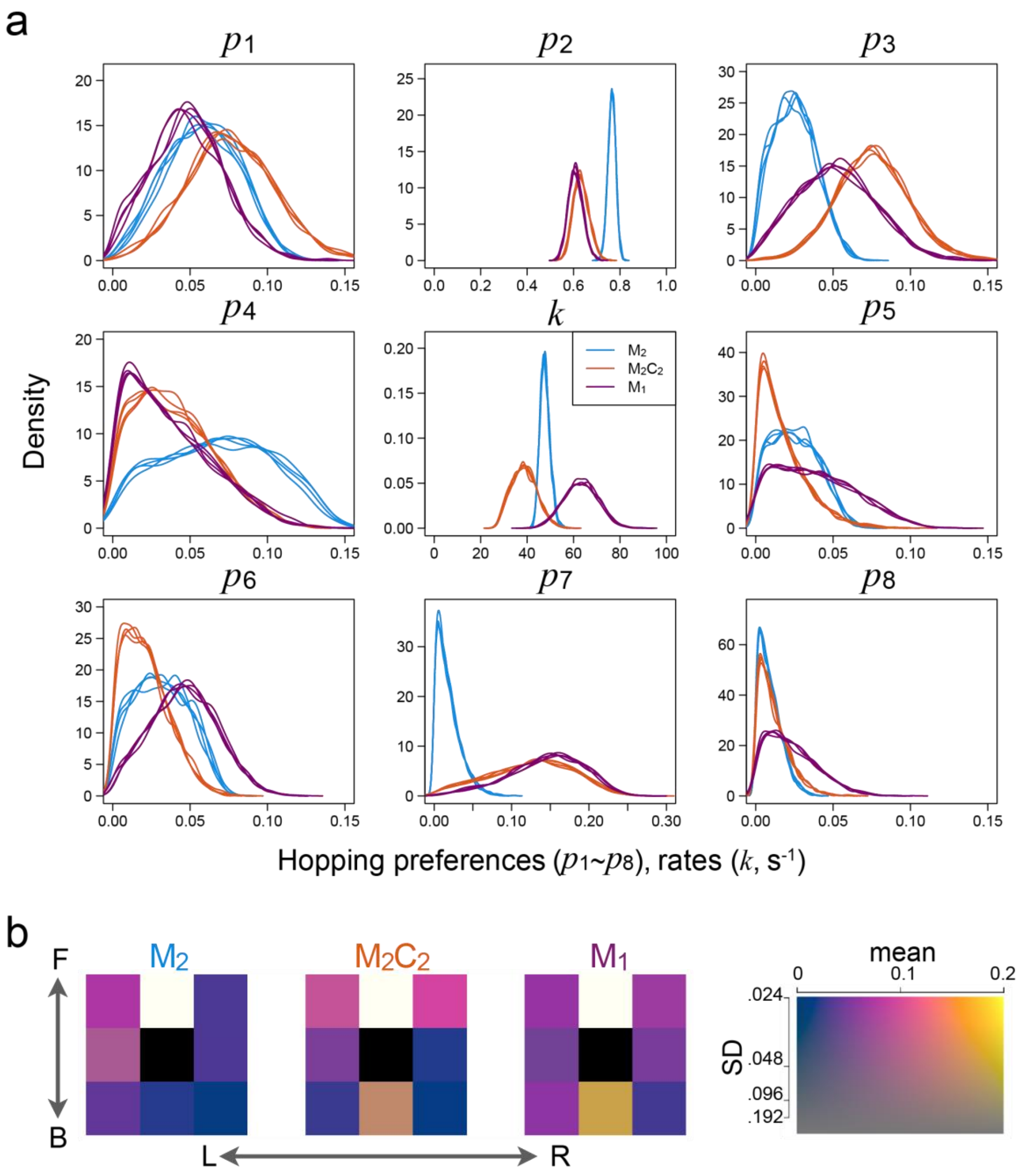

Supplementary Fig. 6: Inference of the directional preferences of the stochastic motion by MKLP1 on the microtubule lattice surface assuming 14-protofilament microtubules.

a, b The results of the Bayesian inference of the hopping rates and preferences (same as in Fig. 5), assuming n = 14. **a** The posterior probability distributions of the overall hopping rate (k) and preferences ($p_1 \sim p_8$, see Fig. 5a) were presented as the density distributions of the four chains of sampling for each construct. **b** The means and standard deviations of the hopping preferences were graphically represented with a color scale shown on the right. The strong bias towards left and forward observed in M_2 was lost in M_2C_2 and M_1 (compare them on p_3 (forward-right) and p_7 (straight backward)). Note that the probability of hopping to the straight forward site (p_2) is much higher than other probabilities and thus it appears saturated (white).

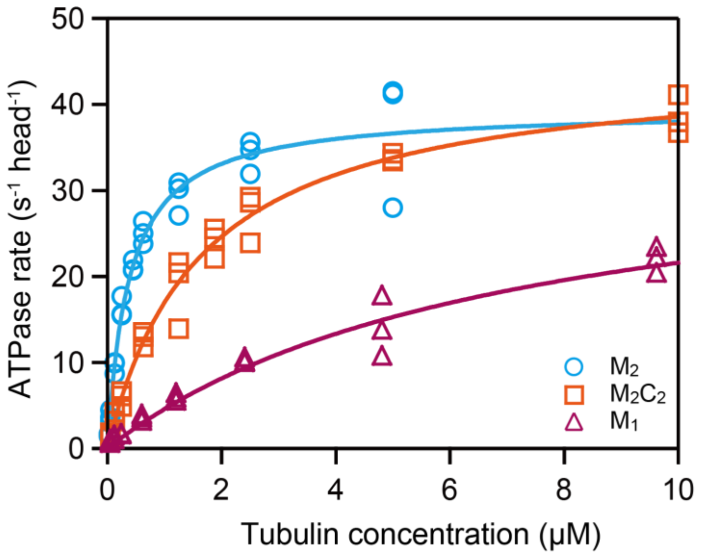

Supplementary Fig. 7: Microtubule-stimulated ATPase activities of MKLP1 constructs.
The microtubule-stimulated ATPase activities were measured from three independent experiments. Maximum of ATP turn-over rate (k_{cat}) and Michaelis constant for microtubule (K_M) were obtained fit by the Michaelis-Menten equation. Rates are expressed in terms of per second per head. Microtubule concentration is expressed as tubulin dimer concentration.

Supplementary Table 1. Summary of inference of the mode of motion

	M_2	M_2C_2	M_1
Number of trajectories	36	47	47
Displacements per trajectory (mean ± SD)	122 ± 53	165 ± 104	133 ± 60
Total displacements	4387	7737	6266
Trapped displacements	241	681	609
Proportion of trapped displacements	5.5%	8.8%	9.7%
Trajectories with trapped segments	17 (47 %)	25 (53 %)	19 (40 %)

Results of the inference of the mode of motion by hidden Markov model (Fig. 4 a–g).
