# Supplementary material for: CYK4 relaxes the bias in the off-axis motion by MKLP1 kinesin-6
Source: Commun Biol. 2021 Feb 10;4:180. doi: 10.1038/s42003-021-01704-2 (PMC7876049; doi:10.1038/s42003-021-01704-2)
Supplement: Supplementary file 6 — Description of Supplementary Files [file 42003_2021_1704_MOESM6_ESM.pdf]

Description of Additional Supplementary Files

File name: Supplementary Movie 1

Description: The M_2-coated bead movements on the suspended microtubule. The movie shows an Alexa488-biotin-labeled microtubule suspended straight between parallel walls via a streptavidin-biotin interaction for the first ~2 seconds (0.1 s intervals, ×1 actual speed, GFP-3035C filter set, Semrock), and an M_2-driven helical motion for the remaining ~5 seconds (0.1 s intervals, ×6 actual speed, G-2A filter set, Nikon). The image is 6.7 μm high and 34 μm wide.

File name: Supplementary Data 1

Description: Data used to generate the charts and graphs in the main figures.
